# A caspase-2-RFXANK interaction and its implication for MHC class II expression

**DOI:** 10.1038/s41419-017-0144-y

**Published:** 2018-01-23

**Authors:** Jeremy Forsberg, Xinge Li, Birce Akpinar, Roger Salvatori, Martin Ott, Boris Zhivotovsky, Magnus Olsson

**Affiliations:** 1 Division of Toxicology, Institute of Environmental Medicine, Karolinska Institutet, Stockholm, Sweden; 2 Present Address: Science for Life Laboratory, Division of Translational Medicine and Chemical Biology, Department of Medical Biochemistry and Biophysics, Karolinska Institutet, Stockholm, Sweden; 3 Department of Biochemistry and Biophysics, Stockholm University, Stockholm, Sweden; 4 Faculty of Medicine, Lomonosov Moscow State University, Moscow, Russia

## Abstract

Despite recent achievements implicating caspase-2 in tumor suppression, the enzyme stands out from the apoptotic caspase family as a factor whose function requires further clarification. To specify enzyme characteristics through the definition of interacting proteins in apoptotic or non-apoptotic settings, a yeast 2-hybrid (Y2H) screen was performed using the full-length protein as bait. The current report describes the analysis of a captured prey and putative novel caspase-2 interacting factor, the regulatory factor X-associated ankyrin-containing protein (RFXANK), previously associated with CIITA, the transactivator regulating cell-type specificity and inducibility of MHC class II gene expression. The interaction between caspase-2 and RFXANK was verified by co-immunoprecipitations using both exogenous and endogenous proteins, where the latter approach suggested that binding of the components occurs in the cytoplasm. Cellular co-localization was confirmed by transfection of fluorescently conjugated proteins. Enhanced caspase-2 processing in RFXANK-overexpressing HEK293T cells treated with chemotherapeutic agents further supported Y2H data. Yet, no distinct differences with respect to MHC class II expression were observed in plasma membranes of antigen-presenting cells derived from *wild type* and *caspase-*2^−/−^ mice. In contrast, increased levels of the total MHC class II protein was evident in protein lysates from caspase-2 RNAi-silenced leukemia cell lines and B-cells isolated from gene-targeted mice. Together, these data identify a novel caspase-2-interacting factor, RFXANK, and indicate a potential non-apoptotic role for the enzyme in the control of MHC class II gene regulation.

## Introduction

The caspase family encompasses a group of endopeptidases regulating cell death and inflammation. While the inflammatory caspases control the production of active pro-inflammatory cytokines and thereby promote innate immune responses, apoptotic caspases target a vast number of protein substrates, ultimately leading to cell fragmentation and elimination. Mechanisms conducting catalytic maturation of apical caspase zymogens, following signal transductions that promote their recruitment into macromolecular complexes, have been described^1^. The interactomes for caspases -8 and -9 support a conceptual view implicating these enzymes in extrinsic and intrinsic apoptosis, respectively. Activation of caspase-2 is controlled by the PIDDosome complex, involving PIDD1 (p53-induced death domain protein 1) and the adapter protein RAIDD (RIP-associated ICH1/CED3-homologous protein with a death domain), formed by homotypic DD (death domain) and CARD (caspase activation and recruitment domain) interactions^2,3^. Alternative, PIDD-independent, mechanisms mediating caspase-2 activation have, however, been described^4–6^. Apart from RAIDD, identification of caspase-2-interacting factors has so far been limited. Recently, the apoptosis inhibitor 5 (API5/AAC11) was reported as an inhibitor of caspase-2 activation owing to the obstruction of CARD-mediated zymogen dimerization^7^. The pro-apoptotic caspase adapter protein (PACAP) was identified by Y2H and verified as a caspases -2 and -9 binding factor with apoptosis-promoting properties^8^. Correspondingly, cyclin D3 seems to potentiate caspase-2 activation during S-phase transition, suggesting a defined link between proliferation and cell death^9^.

Although the caspase-2 function in apoptosis signaling is apparently less restricted than that of caspases -8 and -9, recent achievements suggest that tumor cells lacking caspase-2 frequently suffer from traits relating to genomic instability, especially aneuploidy, thereby proposing an involvement of the protease in the elimination of tumorigenic cells^10,11^. Accordingly, caspase-2 harbors tumor-suppressing properties following oncogenic pressure^12–15^. This function might involve the induction of cell cycle arrest in response to centrosome aberrations^16^, depending on p53 signaling occurring as a consequence of PIDDosome-activated caspase-2, or caspase-2-mediated activation of the BH3-only protein BID (BH3 interacting domain death agonist) and cell death signaling when p53 is lacking in cells facing genomic instability^16,17^.

In addition to the regulation of apoptosis and inflammation, various caspases also fulfill non-apoptotic functions, often associated with different aspects of embryonic development^18^. For example, caspase-8-mediated inactivation of serine/threonine kinase receptor-interacting proteins 1 or 3 (RIPK1, RIPK3) may be required to protect cells from necroptosis^19^, and thereby supports myelomonocytic differentiation into macrophages^20^. Caspase-3, on the other hand, has been implicated in the negative regulation of erythropoiesis via cleavage of the transcription factor GATA-1^21^. Importantly, Fas ligation under these conditions blocked erythroid cell expansion and differentiation without triggering apoptosis. In addition, regulated activation of caspase-3 in various brain cell types can serve as a part of critical cell functions, such as providing a stimulus for microglia to enter a pro-inflammatory phenotype in the absence of death^22^. Several lines of evidence also suggest that caspase-2 may function in processes distinct from apoptosis and cell death-related manifestations. For instance, caspase-2 has been described as a negative regulator of autophagy, as a loss of the enzyme increased LC3-I to LC3-II conversion and protein degradation^23^. In addition, reduced expression of FoxO1 and FoxO3a in old caspase-2-deficient mice, manifested in decreased activities of glutathione peroxidase (GSH-Px) and superoxide dismutase (SOD), served as an explanation for free radical-induced cell damage in these animals and implicated the enzyme in features of antioxidant gene regulation^24^. Further investigations are, however, required for a more complete insight into how caspase-2 function contributes to these processes.

CIITA (class II, major histocompatibility complex transactivator) serves as the “master control factor,” directing the expression of HLA genes (*HLA-DR*, *HLA-DP*, and *HLA-DQ*) in antigen-presenting cells (APC: B-lymphocytes, dendritic cells, macrophages). The heterotrimeric RFX complex, composed of RFXANK, RFX5 (regulatory factor-5) and RFXAP (regulatory factor X-associated protein), X2BP, and NF-Y, ensures coordinated transcription by cis-acting MHC class II (MHC II) promoter motifs (W (S or Z), X1, X2, and Y boxes)^25^. Defects in CIITA or RFX complex factors are reflected in four complementation groups (A–D) of MHC II deficiency (bare lymphocyte syndrome), an autosomal recessive disorder characterized by severely compromised CD4^+^ T-lymphocyte responses. Patients in group B possess RFXANK mutations and MHC II expression is restored by reintroduction of the *wt* gene in corresponding cell lines^26^. Of note, cell type specific promoter usage controlling expression of CIITA leads to the inclusion of a CARD-domain in the CIITA splice isoform 1, expressed in dendritic cells and macrophages, a structural motif also found in caspase-2^27^.

In an unbiased methodological approach, we made an attempt to expand the knowledge of caspase-2 function by means of identification of interacting factors. We found that cytosolic caspase-2 binds to the ankyrin repeat domain of RFXANK. Although no alteration of MHC II was detected in plasma membranes of antigen-presenting cells (APC) from non-exposed caspase-2-deficient mice, an upregulation could be seen in protein lysates from *caspase-*2^−/−^ B-cells, as well as in lysates from acute monocytic leukemia (AMoL) and acute myeloid leukemia (AML) cell lines, when caspase-2 was stably suppressed by RNAi. Therefore, we suggest a novel function for caspase-2, not seemingly associated with any cell death-related mechanism, in the regulation of MHC II protein abundance.

## Results

### Identification of an interaction between caspase-2 and RFXANK by yeast 2-hybrid assay

**Fig. 1:**
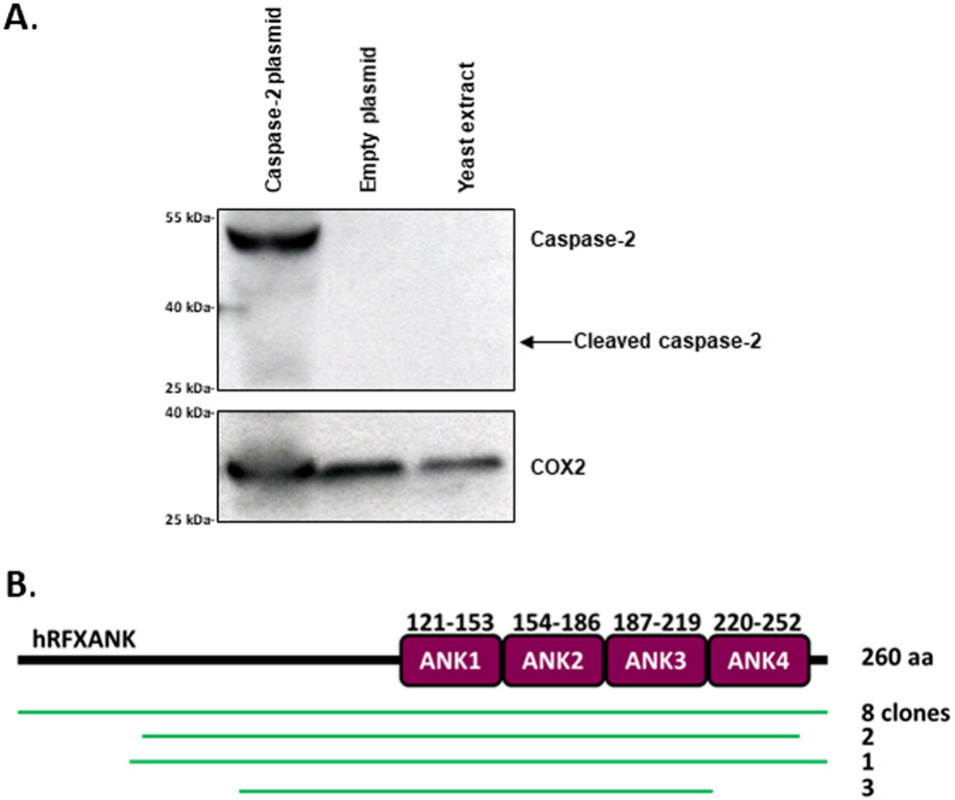
Identification of interaction partners of caspase-2 using Y2H assay **a** The caspase-2 bait construct used in the screening, together with a control, was expressed in *Saccharomyces cerevisiae* followed by analysis with Western blot, in order to confirm its validity. COX2 was used as a control for equal loading. The arrow indicates where cleaved caspase-2 would have appeared when separated on a gel, while using anti-caspase-2 (BD611023) for detection. **b** Representation of hits yielded from the Y2H screening, corresponding to the regions of hRFXANK. All hits overlapped the first three ankyrin repeats of the protein.

Caspase-2 is described as a multifaceted enzyme, implicated in selected apoptotic signaling pathways, as well as in processes serving to avoid aneuploidy and tumorigenic cellular states^28,29^. In an unbiased experimental approach, aimed at detecting caspase-2 protein interactions, which could serve as further elucidation of molecular mechanisms that have been assigned to the enzyme, we performed a Y2H assay using a full-length caspase-2 wild-type construct as bait to screen a randomly primed human lung cancer cell line (A549, H1703, H460) cDNA library (83 million clones, 8-fold the complexity of the library). As the *caspase-2* gene harbors several putative in-frame start codons, the cDNA used as bait was synthesized according to the reported identification of its preferred translation initiation site^30^. Transfection of the bait construct in yeast cells resulted in caspase-2 expression, as verified in SDS-PAGE using a specific antibody targeting the human enzyme (Fig. 1a). No processed fragments of the expressed caspase-2 construct were observed in yeast protein lysates, indicating that any prey proteins might interact with the full-length, inactive enzyme. Notably, the Y2H readout only revealed three high-confidence protein interaction hits and none of the proteins formerly reported to interact with caspase-2, such as PACAP, cyclin D3, API5/AAC11, and RAIDD^2,7–9^, were detected, not even among preys with low or moderate confidence in their bait interaction. Very high confidence in the interaction was, on the other hand, revealed between the caspase-2 bait and the full-length protein, as well as peptides, expressed from a total of 14 cDNA clones with complete homology to the RFXANK (regulatory factor X-associated ankyrin-containing protein; GenBank ID (NCBI): 523498339) splice variant 1 (NM_003721.3) (Fig. 1b). The RFXANK gene is located on 19p13.11 and the corresponding transcription variant 1 translates into a 260 amino acid protein, whose most prominent signature is a protein–protein interaction region consisting of four ankyrin repeats^31,32^. Importantly, partial RFXANK cDNAs, generating truncated protein variants binding to caspase-2 in the Y2H screen, suggested that ankyrin repeats 1–3 or potential upstream motifs were sufficient for the interaction indicated (Fig. 1b).

### Validation of the caspase-2-RFXANK interaction by co-immunoprecipitation and ICC

**Fig. 2:**
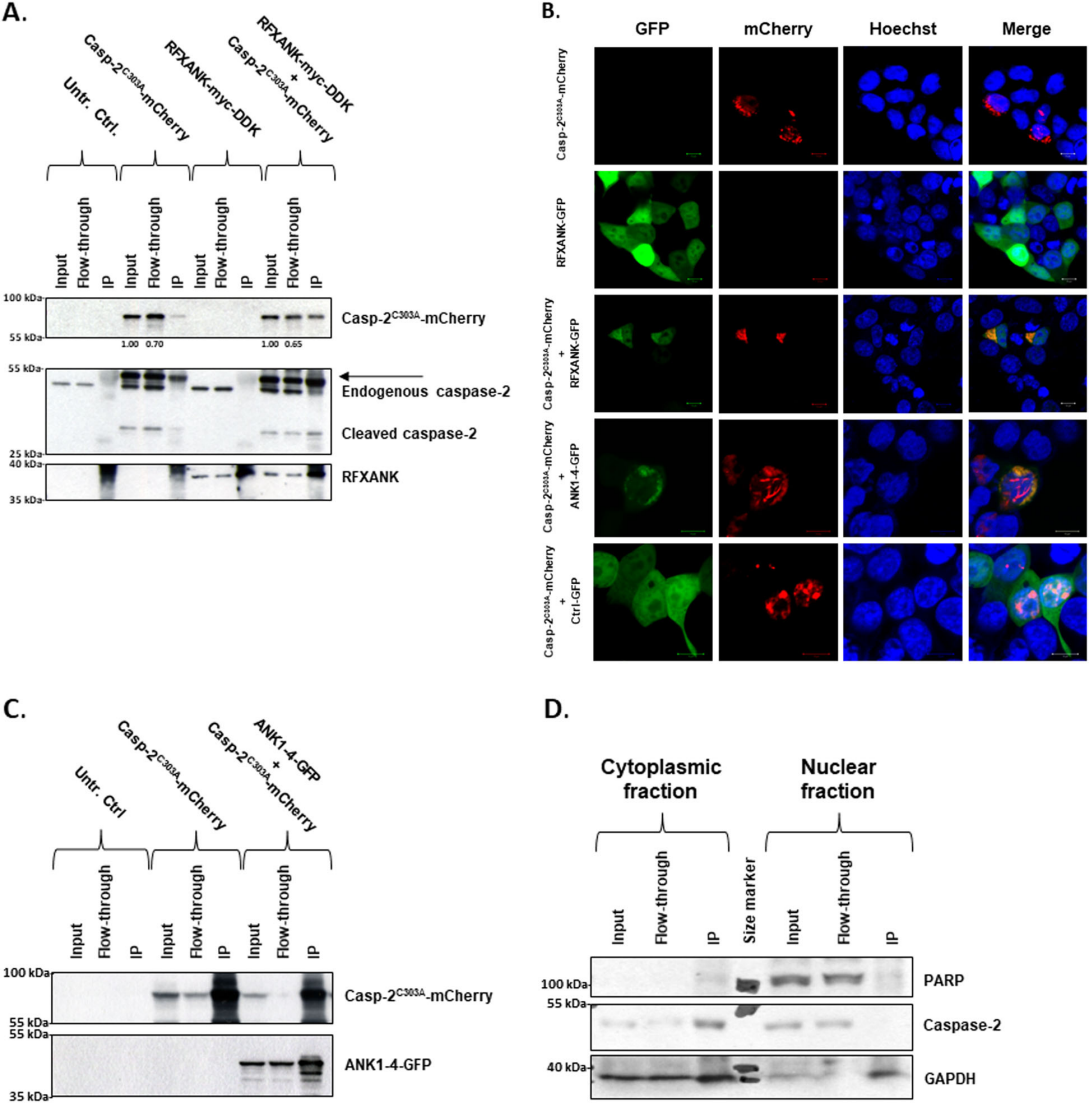
Validation of the caspase-2-RFXANK interaction using co-immunoprecipitation and ICC **a** Co-immunoprecipitation of HEK293T cells expressing Casp-2^C303A^-mCherry and/or RFXANK-myc-DDK. Proteins were captured using anti-RFXANK antibodies and analyzed with Western blot. The relative densities of input vs. flow-through, based on the mean from three individual experiments, are displayed under the Casp-2^C303A^-mCherry blot. The arrow indicates cleaved Casp-2^C303A^-mCherry. **b** Immunofluorescent staining of HEK293T cells expressing Casp-2^C303A^-mCherry (red), and/or RFXANK-GFP (green) or ANK1-4-GFP (green). Hoechst was used to stain nuclei (blue). Bars correspond to 10 µm. **c** Co-IP of HEK293T cells expressing Casp-2^C303A^-mCherry and ANK1-4-GFP. Proteins were captured using anti-RFP antibodies and analyzed with Western blot. **d** Co-IP on endogenous protein levels in OCI-AML2 cells, post-fractionation with digitonin. Proteins were captured using anti-RFXANK antibodies and analyzed with Western blot. Note that the bands of the IP samples in the GAPDH blot correspond to the light chain of the capture antibody. These were difficult to separate from GAPDH on an 8% gel.

In order to validate the interaction between caspase-2 and RFXANK, as suggested by the Y2H screen, we performed co-immunoprecipitation (co-IP) of HEK293T cell lysates after overexpression of RFXANK-myc-FLAG and a catalytically inactive caspase-2 fused to mCherry (Caspase-2^C303A^-mCherry). Through immobilization of RFXANK on magnetic beads, using an RFXANK-specific antibody, Caspase-2^C303A^-mCherry could readily be detected in precipitates following co-expression. Since the antibody used was also able to capture endogenous RFXANK, a small amount of Caspase-2^C303A^-mCherry could be detected even without being transfected with RFXANK-myc-FLAG. Densitometry of the bands, based on the mean from three replicates of the experiment, showed that the relative density decreased in flow-through samples, compared with input (Fig. 2a). Moreover, apart from pull-down of full-length recombinant caspase-2, two processed fragments were detected in the co-IPs (Fig. 2a and Supplementary Figure 1 and 2A). These bands probably arise due to partial processing of the ectopic material. A weak signal from endogenous full-length caspase-2 was observed in the co-transfected sample (Fig. 2a). The absence of caspase-8 in immunoprecipitates support interaction specificity (Supplementary Figure 1). The same experimental setup was also carried out while using the mCherry-N1 control vector instead of Caspase-2^C303A^-mCherry, in which no interaction was observed (Supplementary Figure 2B). In reverse experimental conditions, where immobilization of mutant caspase-2 was accomplished by using a specific antibody against red fluorescent protein (RFP), pull-down of ectopic RFXANK could be identified with Western blotting (Supplementary Figure 1A). Since available antibodies did not work for staining endogenous caspase-2 by immunofluorescence, co-localization studies in HEK293T cells were accomplished using ectopic expression of RFXANK and caspase-2^C303A^, tagged with GFP and mCherry, respectively. Confocal microscopy, using the overexpression approach described, supported IP data showing co-localization of caspase-2 and RFXANK, mainly in cytosolic compartments of HEK293T cells (Fig. 2b). Ectopic RFXANK is ubiquitously localized in HEK293T cells. CARD harbors homotypic interaction properties. Consequently, overexpressed caspase-2 can form dense fibrillary or punctated complexes in the nuclei or in the cytoplasm. Due to the ever-present cell localization of RFXANK in the experimental conditions used, co-localization of the ectopic proteins was seen in all transfected cells. The structures indicated in Fig. 2b were less common and could be found in 5–10% of cells investigated. In comparison to ectopic RFXANK, the endogenous protein is more restricted to the nuclei. Co-localization of endogenous RFXANK and ectopic caspase-2 was also detected mainly in this intracellular compartment (Supplementary Figure 3). As prey clones in the Y2H screen suggested that the ankyrin repeat domain was important for the protein interaction, a co-localization study using the expression of a truncated RFXANK containing ankyrin repeats 1–4 was performed. As the expression of the ankyrin repeat 1–4 construct overlapped with caspase-2 equally well as the full-length RFXANK protein, we concluded this domain to be sufficient for the interaction to occur (Fig. 2b). In support, the caspase-2^C303A^-mCherry was also able to pull down ectopic ankyrin repeats 1–4 upon overexpression in HEK293T cells (Fig. 2c). Finally, it was observed that the caspase-2-RFXANK interaction occurred in the cytoplasmic compartment, as verified by co-IP of endogenous caspase-2 using a specific RFXANK antibody and fractionated acute myeloid leukemia OCI-AML2 cell lysates (Fig. 2d). In summary, these data strongly indicate a cytoplasmic interaction between caspase-2 and RFXANK in non-exposed cells.

### RFXANK overexpression in HEK293T cells facilitates proteolytic caspase-2 processing in response to 5-fluorouracil and doxorubicin

**Fig. 3:**
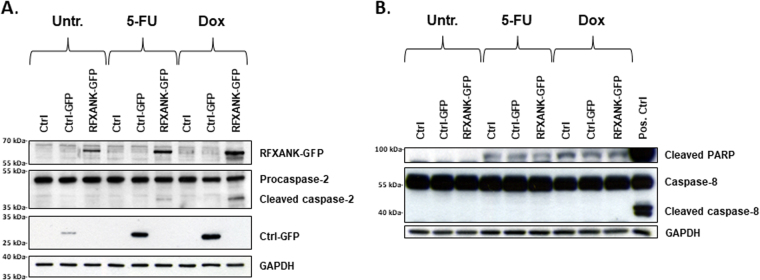
Increased levels of RFXANK facilitate caspase-2 activation following treatment with chemotherapeutic agents **a**, **b** RFXANK-GFP and Ctrl-GFP were expressed in HEK293T cells, followed by 40 h treatment with either 378 µM 5-fluorouracil (5-FU) or 2 µM doxorubicin (Dox). Results were analyzed with Western blot, using GAPDH as control for equal loading. In order to visualize caspase-8 and cleaved PARP, the same samples used in Fig. 3a were analyzed on a separate gel, as they would otherwise partly overlap with the other proteins during the detection step. **b** In conjunction with this, a positive control for cleaved caspase-8 and cleaved PARP was added.

The current close proximity model suggests that apical caspase zymogens possess intrinsic enzymatic activities, which allow for auto-processing in response to clustering at specific protein platforms. Thus, we hypothesized that any caspase-2-interacting proteins at high cellular concentration would facilitate enzyme self-processing under apoptotic conditions. Therefore, RFXANK-GFP was overexpressed in HEK293T cells and subsequently treated with 5-fluorouracil (5-FU) or doxorubicin (Dox). Indeed, in contrast to non-transfected cells and vector controls, ectopic RFXANK accelerated caspase-2 processing in treated cells (Fig. 3a). As indicated by PARP cleavage, detected at equal levels in treated samples, regardless of overexpressed RFXANK, and the absence of caspase-8 processing in these samples, no enhancement of overall apoptosis was observed in response to the combined RFXANK overexpression and chemotherapeutic treatment (Fig. 3b). Since protein overexpression is a highly artificial model, it is important to emphasize that the described data do not necessarily indicate the involvement of RFXANK in apoptotic signaling but rather support interaction data generated in Y2H, immunofluorescence and co-IP experiments.

### Caspase-2 deficiency does not affect MHC class II cell surface expression in healthy mice

**Fig. 4:**
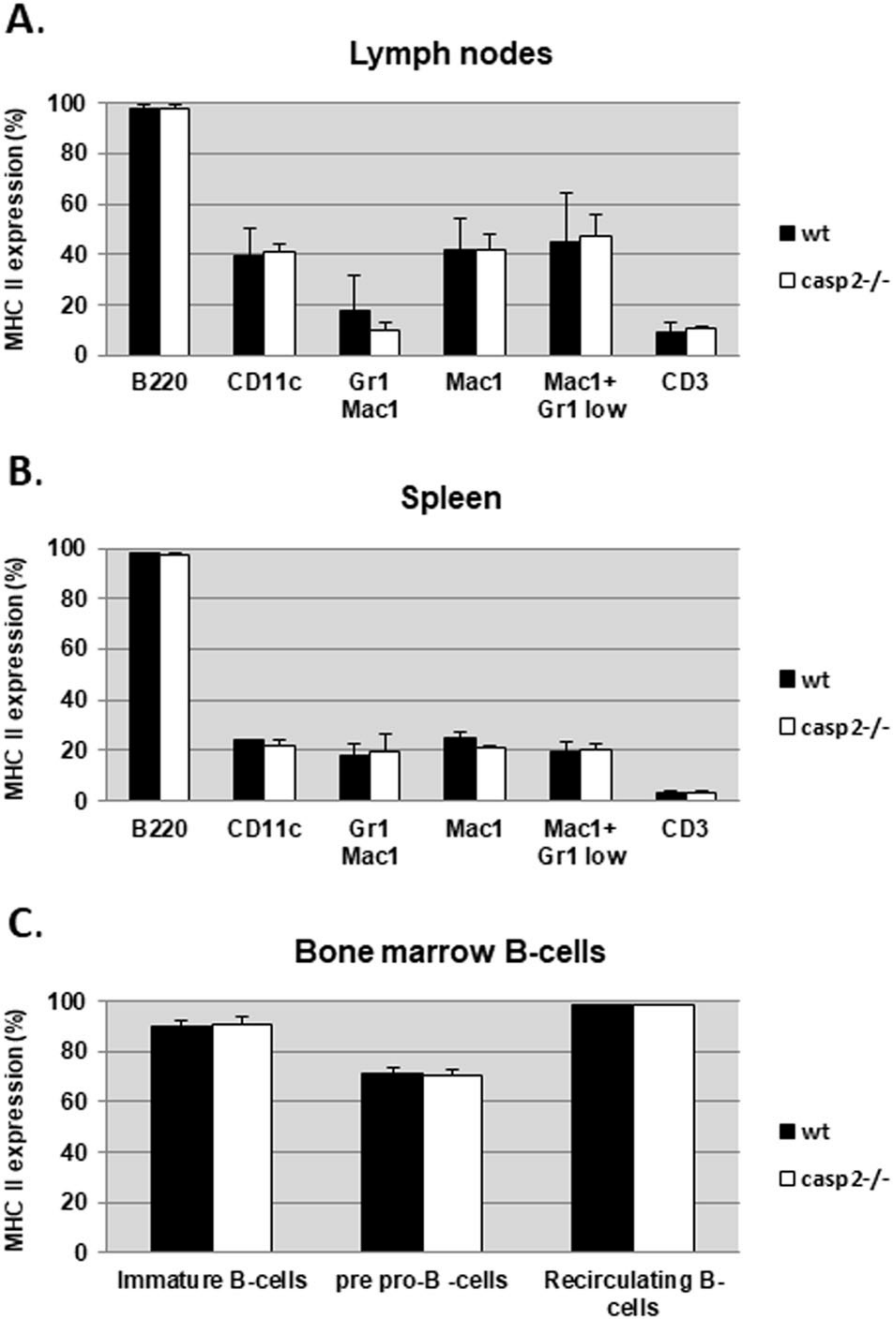
The outcome of caspase-2 deficiency on MHC II expression in murine tissues Surface expression of MHC II was analyzed with flow cytometry on several subsets of blood cells, originating from lymph nodes (**a**), spleen (**b**) and bone marrow (**c**), from three individual mice of *wt* and *caspase2*^−/−^ genetic backgrounds, respectively. Results shown are the mean MHC II expression in the different cell types.

As a component of the CIITA complex, RFXANK is so far exclusively associated with positive regulation of MHC class II gene transcription. Therefore, we made an attempt to investigate whether caspase-2, as a binding partner of RFXANK, also participates in the transcriptional control of these particular genes. For this purpose, cells from primary and secondary lymphoid organs, including lymph nodes, spleen, and bone marrow, were isolated from control and *caspase-2*^−/−^ mice, which was followed by flow cytometry to assess MHC class II (I–E) expression on myeloid and lymphoid cell lineages using specific antibodies (Fig. 4a–c). In summary, irrespectively of cell lineage isolated from lymph nodes (Fig. 4a) and spleen (Fig. 4b), including B220+ B-cells, CD3+ T-cells, CD11c^+^ dendritic cells, and Mac1^+^ Gr1^+^ macrophages, similar levels of MHC II cell surface expression were detected in wild type control and *caspase-2*^*−/−*^ samples. The same conclusion was drawn when analysis of bone marrow-derived IgM- pro/pre-B, immature IgM+D- and recirculating IgM+D+ B-cells was performed (Fig. 4c). Thus, caspase-2 seems not to be involved in the regulation of MHC II cell surface expression in healthy mice.

### Caspase-2 functions as a negative regulator of MHC class II gene expression

**Fig. 5:**
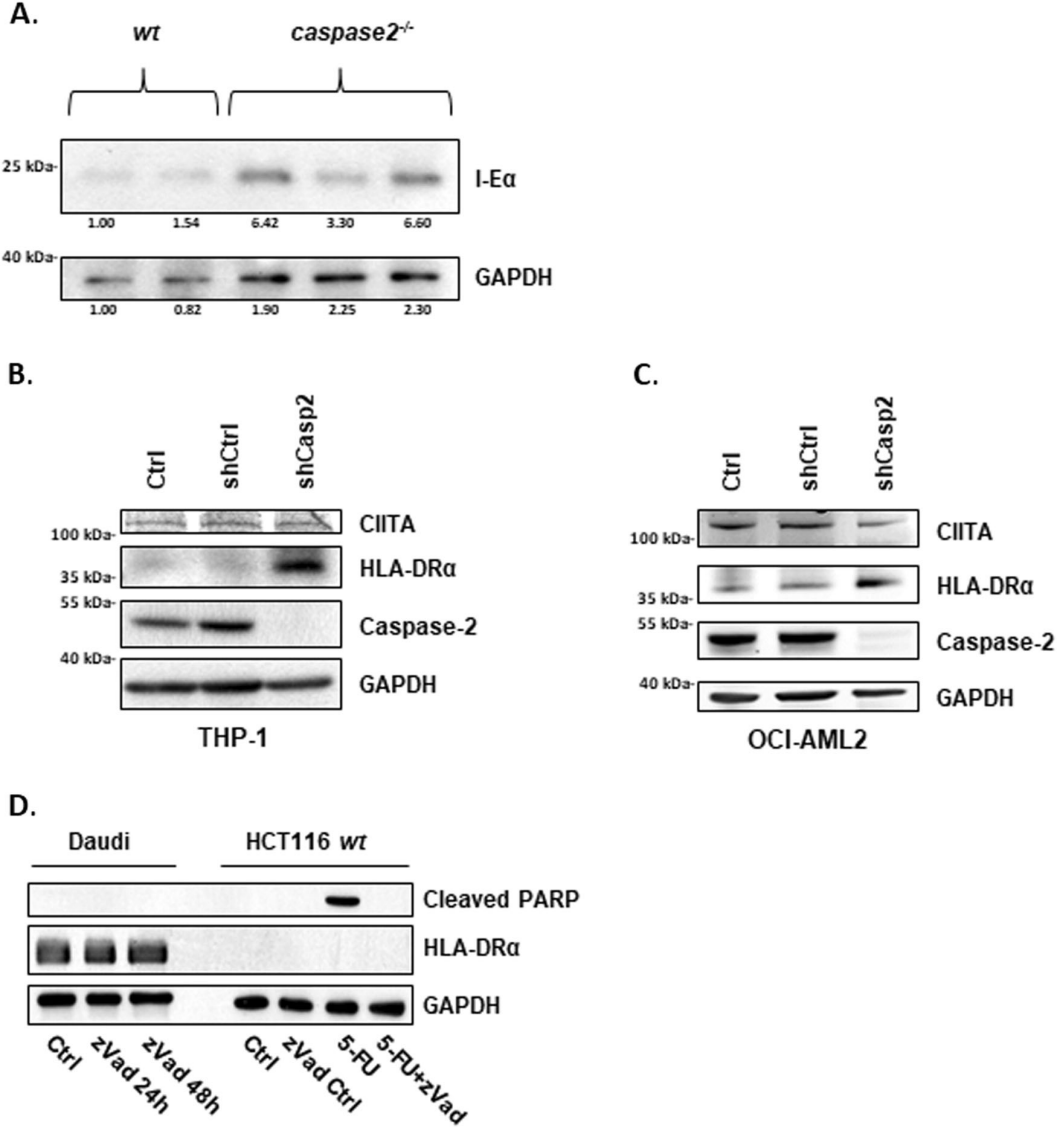
Western blot analysis on total levels of MHC II in human and murine blood cells **a **Total levels of the I-Eα receptor (the murine homologue of HLA-DRα) from *wt* and *caspase-2*^−/−^ murine B-cells. Results presented are from two *wt* and three *caspase-2*^−/−^ animals. The values refer to the relative density of *wt* vs. *caspase-2*^−/−^ samples with respect to I-Eα and GAPDH. Levels of HLA-DRα were assessed in the human leukemic cell lines THP-1 (**b**) and OCI-AML2 (**c**) cell lines after shRNA-mediated knockdown of caspase-2. **d** The impact of the proteolytic activity of caspases on HLA-DRα expression was assessed in Daudi cells by treating cells with the pancaspase inhibitor zVAD-fmk. HCT116 cells treated with 5-FU and zVAD-fmk demonstrated the potency of the inhibitor.

Since a multitude of cellular processes are involved in the processing of antigen, loading on and transport of MHC II, leading to antigen presentation on the plasma membranes of APCs^33^, we followed up the flow cytometric study by investigating whether the total amount of I-Eα and HLA-DRα is affected in isolated mouse *caspase-2*^*−/−*^ B-cells and caspase-2-silenced human leukemia cell lines, respectively. As siRNA transfection per se induces HLA expression in THP-1 cells (Supplementary Figure 4); caspase-2 expression was suppressed by shRNA in the acute monocytic leukemia THP1 and the acute myeloid leukemia OCI-AML2 cell lines. Analysis of protein expression was performed using SDS-PAGE. In all cell models, murine *caspase-2*^−/−^ B-cells (Fig. 5a), THP-1 (Fig. 5b), and OCI-AML2 (Fig. 5c), the absence of caspase-2 generated a distinct upregulation of the MHC II protein when compared to appropriate controls. It is worth to note that in Fig. 5a differences in GAPDH staining were observed despite careful protein concentration analysis. However, since the blot depicts protein levels of individual mice, it is unlikely that the animals would display perfectly equal levels of the same protein, especially when taking into account that GAPDH is a metabolic protein. Together, these data indicate that caspase-2 serves as a negative regulator of total levels of murine I-Eα and human HLA-DRα. However, as verified by flow cytometric analysis (Fig. 4a-c), caspase-2-mediated control of these genes is not reflected on the plasma membranes of APCs in healthy mice. Further, no variation of HLA-DRα was detected in lysates isolated from the Daudi Burkitt’s lymphoma cells treated with the zVAD-fmk pancaspase inhibitor for 72 h, indicating that proteolytic activity of caspase-2, or of other caspases, does not interfere with HLA expression (Fig. 5d). The effectiveness of zVAD-fmk was demonstrated in HCT116 cells, completely blocking PARP cleavage upon treatment with 5-FU. This finding is in line with our Y2H data, where no caspase-2 processing was observed in transfected yeast cells (Fig. 1a).

## Discussion

The ankyrin repeat is one of the most common amino acid motifs in protein databases, and to date, no function apart from mediating protein–protein interactions has been uncovered. Moreover, the range of biological activities in which ankyrin proteins are implicated is paralleled by the diversity of disparate proteins with which they interact^32^.

Although it is plausible, it has not yet been experimentally verified that the RFXANK ankyrin repeat domain participates in the aggregation of RFX complex factors, or recruitment of CIITA. Caspase-2 constitutively localizes throughout various cell compartments, including the nucleus. Cell overexpression is an artificial system, which might influence correct protein localization. Confocal microscopy co-localization analyses of ectopic proteins might, therefore, be misleading with respect to the cellular compartment in which the interaction occurs. However, IP data using endogenous material localized the caspase-2-RFXANK interaction to the cytoplasm, thereby, disconnecting the latter factor from direct interference with MHC II gene transactivation. Yet, there are other putative mechanisms that can explain the inhibition of MHC II expression by caspase-2. A specific nuclear localization signal (NLS), which can be recognized by the importin α/β heterodimer, has not been identified in RFXANK, and the protein localizes poorly to the nucleus^34^. Analogously to how ß-catenin enters the nucleus by interaction with FoxM1^35^, a piggyback model would provide one explanation for the nuclear translocation of RFXANK. Caspase-2, on the other hand, contains an NLS in the C-terminal CARD but, similarly to other caspases, the protein mainly localizes to the cytosol in most cell lines (Ref.^36^ and our own unpublished observations). Caspase-2 may therefore serve as a cytoplasmic tethering factor for RFXANK, comparable to PARC (p53-associated parkin-like cytoplasmic protein), whose main effect on p53 seems to be cytoplasmic sequestration. Like a decline in cytosolic PARC levels emancipates p53 for subsequent transcription regulatory events^37^, a still unidentified mechanism may break the caspase-2-RFXANK bond and, by doing so, contribute to MHC II gene regulation. Although upregulation of the total MHC II was evident in lysates from caspase-2 RNAi-silenced human leukemia cell lines, and B-cells isolated from gene-targeted mice (HLA-DRα and I-Eα, respectively), no variation of MHC II was evident in FACS analysis of APCs isolated from *caspase-2*^−/−^ mice when compared to cells from control littermates. A reasonable explanation for this inconsistency is that enhanced HLA gene expression is not necessarily reflected in a more frequent exposure of the corresponding proteins on the cell surface. A multitude of regulatory layers are implicated in this process, including APC maturation, antigen loading, protein trafficking, and internalization of peptide-loaded MHC class II molecules^33^. Further experimental efforts are certainly required in order to clarify whether or not caspase-2 contributes to the adaptive T-cell immune response elicited by antigen-presenting MHC II molecules, and whether the lack of the enzyme is an advantage or a disadvantage for the efficiency of the process.

The global target-gene specificity of the CIITA protein is almost exclusively focused on genes implicated in antigen presentation^38^. However, a global survey of target genes connected to the transcriptional activity of RFXANK has not yet been carried out. Bearing in mind that CIITA expression is limited to B-cells, macrophages and dendritic cells while both caspase-2 and RFXANK are ubiquitous proteins (http://www.proteinatlas.org/), it is tempting to speculate that this novel protein interaction, apart from MHC II, is also involved in the control of other types of gene regulatory events.

## Materials and methods

### Cell culture

The HCT116 and HEK293T cell lines were cultured in Dulbecco’s Modified Eagle’s Medium (DMEM), supplemented with 10% heat-inactivated fetal bovine serum and PenStrep (100 U/mL penicillin, 100 mg/mL streptomycin). Daudi and THP-1 cells were cultured in RPMI 1640, supplemented with 10% heat-inactivated bovine serum and PenStrep (100 U/mL penicillin, 100 mg/mL streptomycin). OCI-AML2 cells were cultured in alpha-modified Minimum Essential Medium (MEM), supplemented with 20% heat-inactivated fetal bovine serum and 1% L-glutamine. All cell culture reagents were purchased from GIBCO (Invitrogen). Cells were cultured in a humidified 5% CO_2_ atmosphere at 37 °C and maintained in a logarithmic growth phase for all experiments. Treatments of HEK293T cells with 5-fluorouracil (5-FU, Accord Healthcare Ltd) and doxorubicin hydrochloride (Sigma-Aldrich) were performed according to the concentrations and incubation times indicated in the figure.

### Yeast 2-hybrid analysis

Yeast two-hybrid screening was performed by Hybrigenics Services, S.A.S., Paris, France (http://www.hybrigenics-services.com). The coding sequence for caspase-2 (NM_032982.3) was PCR-amplified and cloned into pB27 as a C-terminal fusion to LexA (LexA-CASP2). The construct was checked by sequencing the entire insert and used as bait to screen a randomly primed human lung cancer cell line (A549, H1703, H460) cDNA library constructed into pP6. pB27 and pP6 derive from the original pBTM116^39^ and pGADGH (Bartel et al., 1993) plasmids, respectively. Eighty-three million clones (8-fold the complexity of the library) were screened using a mating approach with YHGX13 (Y187 ade2-101:loxP-kanMX-loxP, matα) and L40ΔGal4 (mata) yeast strains as previously described^40^. A total of 169 His+ colonies were selected on a medium lacking tryptophan, leucine, and histidine, and supplemented with 5 mM 3-aminotriazole to handle bait auto-activation. The prey fragments of the positive clones were amplified by PCR and sequenced at their 5’ and 3’ junctions. The resulting sequences were used to identify the corresponding interacting proteins in the GenBank database (NCBI) using a fully automated procedure. A confidence score (PBS, for Predicted Biological Score) was attributed to each interaction as previously described^41^.

### Yeast transformation and protein extraction

A single colony of *Saccharomyces cerevisiae* W303a was inoculated from a YPD (1% yeast extract, 2% peptone, 2% dextrose, w/v) plate to a 5 mL YPD liquid culture and grown overnight in a rotary shaker at 170 rpm and 30 °C. The preculture was diluted to OD = 0.1 in YPD and put back onto the rotary shaker. At OD = 1, 1.5 mL of culture was spun down in a tabletop centrifuge, washed with water, resuspended in 1 mL of 0.1 M lithium acetate, and incubated at 700 rpm and 30 °C for 10 min. The cells were then spun down and resuspended in 33% polyethylene glycol, 100 mM lithium acetate, 50 μg of DNA sodium salt from salmon testes, and 0.1 μg of plasmid DNA (pB27). The cells were vortexed and heat-shocked for 45 min at 42 °C and 800 rpm, and then spun down and recovered in 1 mL YPD for 1 h at 30 °C and 1100 rpm. The cells were plated on a selective plate (synthetic defined medium without tryptophan). The total protein amounts were retrieved by performing a Rödel extraction on overnight cultures from individual colonies. Briefly, cells that had been grown in YPD were harvested by centrifugation and resuspended in 250 µL H_2_O. A fresh Rödel mix (741 µL H_2_O, 185 µL 10 M NaOH, 74 µL 14.3 M β-mercaptoethanol) was prepared, from which 50 µL was added to each sample. This was followed by 10 min incubation on ice, before adding 60 µL 72% TCA. Subsequently cells were incubated at −20 °C for 20 min, and then centrifuged at 4 °C for 30 min at 28,000 rcf. The pellets were washed once with acetone before being resuspended in Laemmli buffer and analyzed using SDS-PAGE.

### Immunoprecipitation

HEK293T cells were seeded in petri dishes, using antibiotic-free DMEM medium, to 70% confluency and then transfected with the plasmids outlined in the figures using Lipofectamine® LTX (Invitrogen) according to the manufacturer’s recommendations. For individual transfections, 8 µg of plasmid DNA was used, whereas for the co-transfection, 4 µg of each plasmid was utilized. The final volume after adding the transfection mixtures was 2 mL. After 6 h incubation, cells were detached, washed in PBS, and centrifuged. Pelleted cells were resuspended in 500 µL lysis buffer (30 mM Tris-HCl pH7.5, 120 mM NaCl, 1% glycerol, 0.5% NP-40, supplemented with Complete Protease Inhibitor Cocktail and PhosSTOP (Roche Diagnostics)), and subsequently left on ice for 30 min, followed by centrifugation at 13,684*g* for 20 min. The lysates were then incubated with either anti-RFXANK- or anti-RFP-coated Dynabeads Protein G (Thermo Scientific) overnight at 4 °C, under constant rotation. Each sample was then placed in a DynaMag2 (Life Sciences), allowing the bead complexes to be separated from the unbound proteins (i.e., flow-through). Subsequently, each sample was washed with lysis buffer for 3 × 5 min under rotation at room temperature. Finally, each complex was resuspended in 30 µL 1x Laemmli buffer and analyzed by Western blotting.

### Cellular fractionation

OCI-AML2 cells were harvested and washed with PBS, followed by 5 min of centrifugation. The pellet was then resuspended in 100 µL/million cells of a fractionation buffer (150 mM KCl, 1 mM MgCl_2_, 0.2 mM EGTA, 5 mM Tris, and 0.01% digitonin), and left to incubate for 10 min at room temperature. After briefly being vortexed, the cells were centrifuged at 16,000*g* at 4 °C, and the supernatant (cytosolic fraction) was collected. The pellet (nuclear fraction) was resuspended in the NP-40 buffer mentioned above, followed by 30 min incubation on ice. Immunoprecipitation was then carried out as has been described.

### Western blot

Protein concentrations were measured with the BCA assay (Pierce). For immunoprecipitation analyses, only the input samples were measured. All protein samples were analyzed with SDS-PAGE, using either 8 or 12% polyacrylamide gels or 4–15% gradient gels (Bio-Rad). For whole cell lysates, 10–30 µg was loaded, while for immunoprecipitates 30 µg input material and the corresponding volume of flow-through material were applied to the gels. Separated proteins were then electroblotted to nitrocellulose membranes (0.45 µm, Bio-Rad), which were subsequently blocked with 5% (w/v) dry milk in PBS and probed with the primary antibody of interest diluted in PBS containing 1% (w/v) bovine serum albumin (BSA), 0.05% Tween 20 and 0.025% azide at 4 °C, overnight. Membranes were washed in PBS and PBS-Tween20 (0.05%) prior to 1 h of incubation at room temperature with horseradish peroxidase (HRP)-conjugated secondary antibodies. Following repeated washing steps, membranes were revealed by enhanced chemiluminescence (GE Healthcare Biosciences) and exposed to SuperRX X-ray films (Fujifilm Corporation).

### Immunofluorescence

HEK293T cells were seeded on 4 cm diameter glass coverslips (thickness no. 0) and the following day transfected using Lipofectamine® LTX (Invitrogen) according to the manufacturer’s recommendations and the plasmids outlined in the figures. Examination under live cell conditions was performed 24–48 h post-transfection using a Zeiss LSM 510 META confocal laser scanner microscope (Carl Zeiss MicroImaging) and analysis by the accompanying software. Nuclei counterstaining was accomplished using a Hoechst 33342 (1 μg/mL, Thermo Scientific).

### Animal experiments

Organs were harvested from *wild-type* or *caspase-2*^−/−^ C57BL/6 mice, sacrificed according to the legal requirements of the Austrian Animal Law. Red blood cell lysis was performed using a buffer containing 145.6 mM NH4Cl, 0.127 mM EDTA, and 23.8 mM NaHCO3 in spleen and peripheral blood samples.

(1) FACS-sorting B-cells from spleen of *wild-type* or *caspase-2*^−/−^ mice: single cell suspension of splenocytes was stained with the following biotin-conjugated antibodies, Ter119-bio (TER-119), CD3e-bio (145-2C11), TCRβ-bio (H57-597), ɣdTCR-bio (eBioGL3), Mac1-bio (M1/70), NK1.1-bio (PK136), and Gr1-bio (RB6-8C5), for 1 h at 4 °C. After washing cells with PBS, cells were again stained with PE/Cy7-streptavidin (biolegend Lot: B172413) secondary antibody. Viable (DAPI-negative) B-cells (PE/Cy7-negative) were FACS-sorted using a BD ARIA III FACS sorter. Cells were then snap-frozen using liquid N2 and then further processed for immunoblotting.

(2) MHC-II distribution of murine hematopoietic cell subsets of *wild-type* or *caspase-2*^−/−^ mice: Single cell suspensions of different organs were processed for flow cytometry using a BD LSR-Fortessa and the following antibodies (30 min at 4 °C): Peripheral blood: MHC-II-PE (M5/114.15.2), CD19-eF605 (6D5), B220-APC780 (RA3-6B2), IgM-APC (RMM-1), IgD-PerCP/Cy5.5 (11–26 c.2a), and AnnexinV-eF450 (eBioscience 88-8006-74). Lymph nodes and spleen: MHC-II-PE (M5/114.15.2), CD11c-alexa647 (N418), B220-APC780 (RA3-6B2), F4/80-eF605 (BM8), Mac1-FITC (M1/70), CD3-PE/Cy7 (145-2C11), Gr1-PerCP/Cy5.5 (RB6-8C5), and AnnexinV-eF450 (eBioscience 88-8006-74). Bone marrow: MHC-II-PE (M5/114.15.2), CD19-eF605 (6D5), IgM-APC (RMM-1), and AnnexinV-eF450 (eBioscience 88-8006-74).

### Plasmids

The GFP-RFX-B/ANK construct was kindly provided by Professor Jeremy M. Boss (Emory University School of Medicine, Atlanta, USA) and has been described elsewhere^34^. Control pEGFP-N1, pmCherry-N1, and pCMV6-RFXANK (NM_003721) containing C-terminal Myc-DDK were purchased from Clontech and Origene, respectively. The pCaspase-2^C303A^-mCherry was generated through PCR amplification of the pGALL-(HIS3)-caspase-2^C303A^ vector (kindly provided by Professor Christine Hawkins, Murdoch Children’s Research Institute, Parkville, Australia) using the following primers (Invitrogen): forward primer, 5′-ttagatctatgcatcctcatcatcaggaaactctaaaa-3′, and reverse primer, 5′-tgggccctgtgggagggtgtcctgggaacaggtagag-3′. The product was cleaved using BglII and ApaI and ligated into the corresponding sites of pmCherry-N1. Similarly, a GFP vector containing the sequences encoding the four ankyrin repeats (ANK1–4-GFP) was generated using the forward primer, 5′-ttccctcgagatgaccctagactccctgtc-3′, and the reverse primer, 5′-aaggggatcctcggtggtgaggtcagcg-3′. The product was cleaved using Xho1 and BamH1 and ligated into the corresponding sites of pEGFP-N1.

### Antibodies

For Western blotting, the following antibodies were used: anti-RFXANK polyclonal antibody (pAb) (Sigma-Aldrich), anti-caspase-2 (C20) pAb (Santa Cruz Biotechnology), anti-caspase-2 monoclonal antibody (mAb), clone 35 (Becton-Dickinson), anti-glyceraldehyde 3-phosphate dehydrogenase (G3PDH/GAPDH) (Trevigen), anti-HLA-DRα (G-7) (Santa Cruz Biotechnology), anti-CIITA (7–1H) (Santa Cruz Biotechnology), anti-PARP (Roche), anti-cleaved PARP (Cell Signaling), anti-caspase-8 mAb, clone C15 (kindly provided by Professor P.H. Krammer and Dr I. Lavrik, German Cancer Research Center, Heidelberg, Germany), anti-RFP pAb (Rockland Immunochemicals), and anti-GFP (Roche). For antibodies used on murine samples, please see the section relating to animal experiments.

## Electronic supplementary material

Supplementary Figure Legends

Supplementary Figure 1

Supplementary Figure 2

Supplementary Figure 3

Supplementary Figure 4
