# Supplementary material for: A caspase-2-RFXANK interaction and its implication for MHC class II expression
Source: Cell Death Dis. 2018 Jan 23;9(2):80. doi: 10.1038/s41419-017-0144-y (PMC5833739; doi:10.1038/s41419-017-0144-y)
Supplement: Supplementary file 1 — Supplementary Figure Legends [file 41419_2017_144_MOESM1_ESM.docx]

**Supplementary Figure Legends**

**Suppl. Figure 1. Co-immunoprecipitation of HEK293T cells expressing Casp-2^C303A^-mCherry and/or RFXANK-myc-DDK**.

Proteins were captured using either of two different antibodies targeting RFXANK: lanes 1-3 (Atlas Antibodies) and 7-12 (Sigma-Aldrich). Control samples are indicated in the figure.

**Suppl. Figure 2. Caspase-2 interacts with RFXANK**.

(**A**) Co-immunoprecipitation of HEK293T cells expressing Casp-2^C303A^-mCherry and/or RFXANK-myc-DDK. Proteins were captured using anti-RFP antibodies and subsequently analyzed with Western blot. (**B**) Overexpression of RFXANK and an empty mCherry control vector, followed by co-IP using anti-RFP antibodies for capture.

**Suppl. Figure 3. Co-localization of ectopic caspase-2 and endogenous RFXANK in immunocytochemistry.**

HEK293T cells were transfected with Casp-2^C303A^-mCherry (red), while retaining endogenous levels of RFXANK. To detect the latter, anti-RFXANK (Sigma) was used (green). Nuclei were stained with Hoechst (blue). Samples were analyzed using confocal microscopy.

**Suppl. Figure 4. siRNA treatment causes induction of MHC proteins in THP-1 cells.** Macrophage cell line THP-1 was transfected with control and caspase-2 targeting siRNA constructs, and later analyzed with Western blot. GAPDH was used as a control for equal loading. The same samples were analyzed on two different membranes.
