# Supplementary figures and images for: A caspase-2-RFXANK interaction and its implication for MHC class II expression
Source: Cell Death Dis. 2018 Jan 23;9(2):80. doi: 10.1038/s41419-017-0144-y (PMC5833739; doi:10.1038/s41419-017-0144-y)

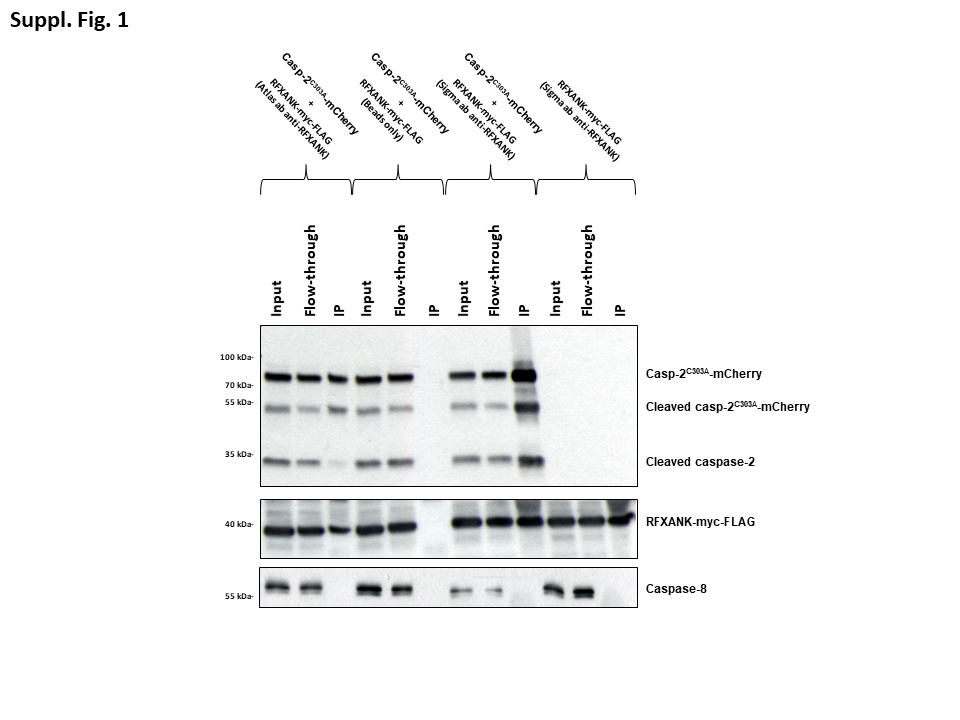

Supplement: Supplementary file 2 — Supplementary Figure 1 [file 41419_2017_144_MOESM2_ESM.tif]

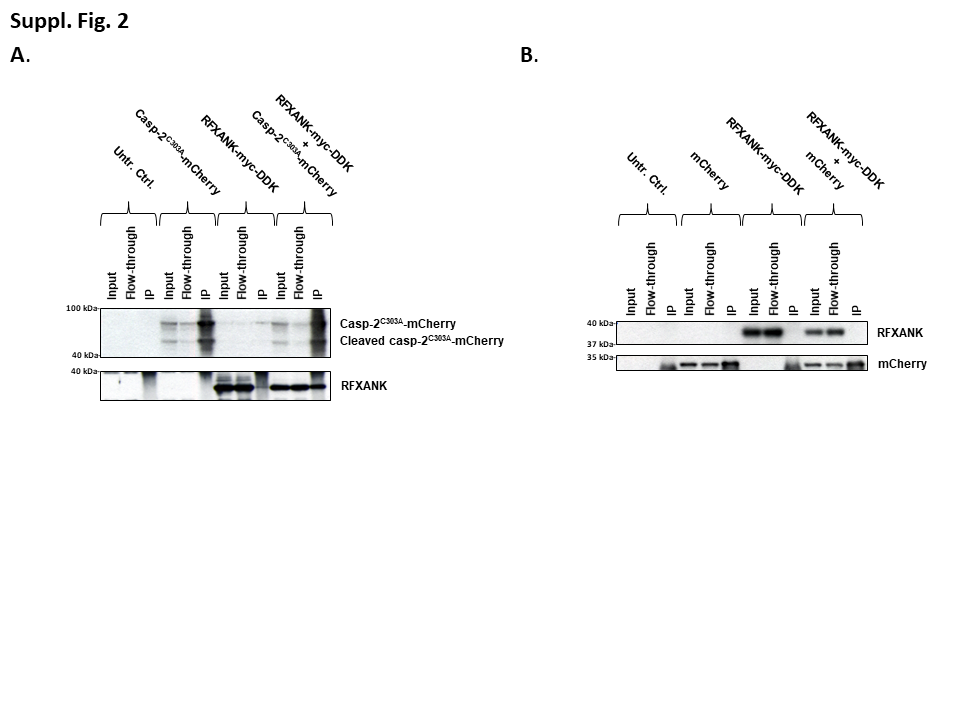

Supplement: Supplementary file 3 — Supplementary Figure 2 [file 41419_2017_144_MOESM3_ESM.tif]

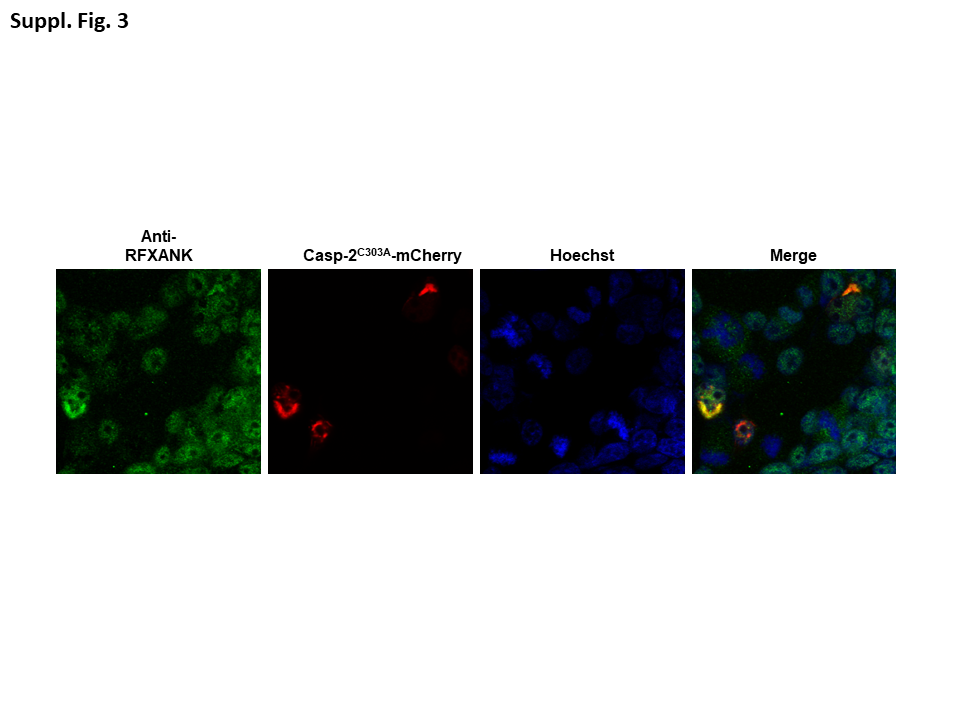

Supplement: Supplementary file 4 — Supplementary Figure 3 [file 41419_2017_144_MOESM4_ESM.tif]

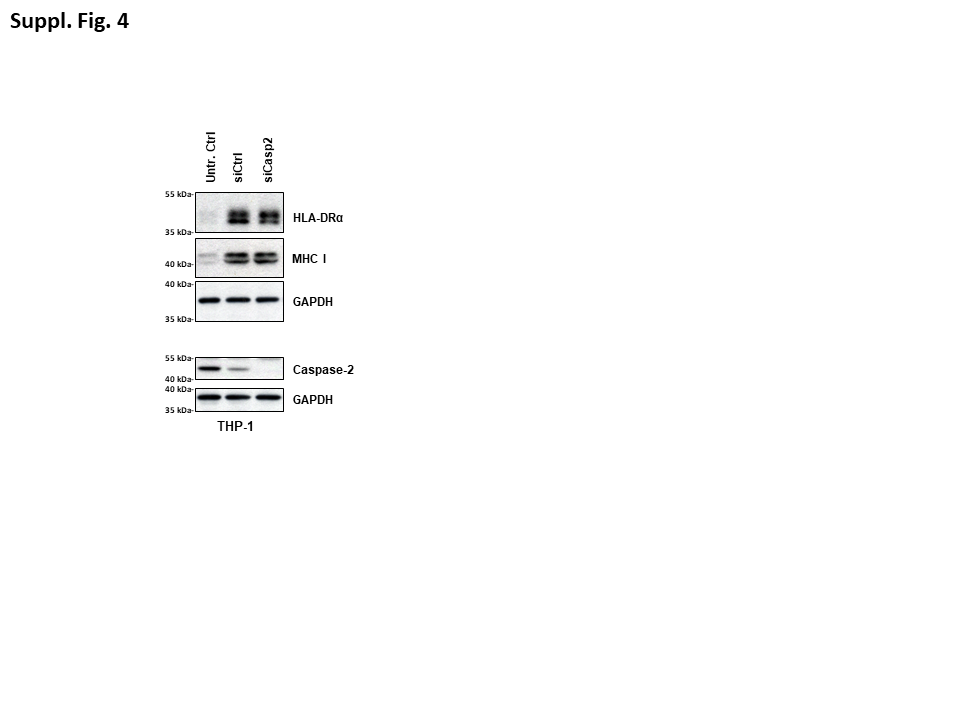

Supplement: Supplementary file 5 — Supplementary Figure 4 [file 41419_2017_144_MOESM5_ESM.tif]
